# Prognostic value of red blood cell distribution width-to-albumin ratio in ICU patients with coronary heart disease and diabetes mellitus

**DOI:** 10.3389/fendo.2024.1359345

**Published:** 2024-09-25

**Authors:** Sheng Chen, Senhong Guan, Zhaohan Yan, Fengshan Ouyang, Shuhuan Li, Lanyuan Liu, Liuer Zuo, Yuli Huang, Jiankai Zhong

**Affiliations:** ^1^ Department of Cardiology, Shunde Hospital, Southern Medical University (The First People’s Hospital of Shunde), Foshan, Guangdong, China; ^2^ Department of Rehabilitation Medicine, Shunde Hospital, Southern Medical University (The First People’s Hospital of Shunde), Foshan, Guangdong, China; ^3^ Department of Pediatrics, Shunde Hospital, Southern Medical University (The First People’s Hospital of Shunde), Foshan, Guangdong, China; ^4^ Department of Ultrasound Medicine, Shunde Hospital, Southern Medical University (The First People’s Hospital of Shunde), Foshan, Guangdong, China; ^5^ Department of Intensive Care Unit, Shunde Hospital, Southern Medical University (The First People’s Hospital of Shunde), Foshan, Guangdong, China

**Keywords:** red blood cell distribution width-to-albumin ratio, coronary heart disease, diabetes mellitus, all-cause mortality, MIMIC-IV

## Abstract

**Background:**

The red blood cell distribution width (RDW)-to-albumin ratio (RAR) has emerged as a potentially valuable prognostic indicator in diverse medical conditions. However, the prognostic significance of RAR in intensive care unit (ICU) patients with coronary heart disease (CHD) and diabetes mellitus (DM) remains uncertain and requires further investigation.

**Methods:**

This study aims to investigate the prognostic significance of RAR in ICU patients with coexisting CHD and DM through a retrospective cohort analysis using data from the Medical Information Mart for Intensive Care IV (MIMIC-IV) database (version 2.2). The study population included patients aged 18 years or older who were diagnosed with both CHD and DM. The primary endpoint was 1-year mortality, and the secondary endpoints included 30-day mortality, 90-day mortality, hospital length of stay (LOS), and ICU LOS.

**Results:**

A total of 3416 patients, of whom 64.64% were male, were included in the study. The 30-day mortality, 90-day mortality, and 1-year mortality were 7.08%, 7.44%, and 7.49%, respectively. After adjusting for confounding factors, multivariate Cox proportional risk analysis demonstrated that high RAR levels were associated with an increased risk of 30-day mortality (HR, 1.53 [95% CI 1.17-2.07], P = 0.006), 90-day mortality (HR, 1.58 [95% CI 1.17-2.13], P = 0.003), and 1-year mortality (HR, 1.58 [95% CI 1.17-2.13], P = 0.003). Furthermore, the restricted cubic spline (RCS) model indicated a linear relationship between RAR and 1-year mortality.

**Conclusion:**

The results suggest that RAR holds potential as a valuable prognostic biomarker in ICU patients with both CHD and DM. Elevated RAR levels were found to be significantly associated with increased mortality during hospitalization, facilitating the identification of individuals at higher risk of adverse outcomes. These findings underscore the importance of incorporating RAR into risk stratification and overall management strategies for ICU patients with coexisting CHD and DM.

## 1 Introduction

Patients in a critical state, admitted to the intensive care unit (ICU), often exhibit intricate conditions with diverse etiologies, leading to a considerable mortality of 8.5% (1). Concurrently, coronary heart disease (CHD) afflicts approximately half of the patient population within the ICU (1). Notably, Diabetes Mellitus (DM) remains a prominent risk factor for CHD (2), contributing significantly to an augmented risk of cardiovascular pathologies due to heightened fasting glucose levels and insulin resistance characteristically associated with DM (3, 4). This relationship amplifies the mortality risk from CHD by two to four-fold (5). The co-morbidity of CHD and DM in ICU patients intensifies the complexity of treatment and resultant mortality. Despite these challenges, there is a notable absence of research dedicated to probing prognostic outcomes of ICU patients burdened with both CHD and DM. Therefore, identifying factors that contribute to mortality within this demographic in a timely manner proves instrumental in enhancing prognostic outcomes and shaping appropriate therapeutic interventions.

Red blood cell distribution width (RDW) serves as a valuable marker for identifying abnormal size distribution of red blood cells (RBCs) in peripheral blood (6). Previous investigations have demonstrated that elevated RDW levels independently contribute to an increased risk of unfavorable prognosis in cardiovascular conditions, including acute myocardial infarction, atrial fibrillation, and aortic aneurysm (7, 8). Similarly, studies have established a correlation between RDW and both mortality and cardiovascular complications in patients with DM (9). Furthermore, heightened RDW levels have been associated with more extensive and intricate coronary artery disease, as well as a greater incidence of cardiovascular events in individuals with DM (10). Collectively, these findings suggest that RDW holds promise as a clinical marker for assessing CHD in the context of DM. However, it is important to acknowledge that RDW levels can be influenced by various factors such as lifestyle habits, renal impairment, or certain medications (7, 11). Consequently, relying solely on RDW level predictions may not provide definitive outcomes.

Albumin, a vital protein that signifies nutritional and inflammatory status, has also emerged as a significant indicator (12). Lower albumin levels have been shown to be negatively correlated with the inflammatory response and are associated with an increased risk of cardiovascular mortality (13, 14). However, the role of serum albumin as a prognostic indicator may also be limited by other factors such as chronic diseases, malnutrition, and inflammation (15). Accordingly, depending exclusively on albumin level predictions is insufficient. Several studies have highlighted that combining RDW and albumin yields more meaningful assessments of disease prognosis. Specifically, Li et al. discovered that high RDW-to-albumin ratio (RAR) levels were linked to increased 90-day mortality in patients with acute myocardial infarction (16). Recent investigations have also indicated the association between RAR and prognosis in patients with DM (17–19). Notably, Huang et al. identified a stronger relationship between RAR and carotid plaque in individuals with CHD and DM (20). This indicates that RAR seems to be a prognostic indicator for patients with CHD and DM. Nevertheless, to our knowledge, previous studies have not evaluated the association between RAR and prognosis in critically ill patients with CHD combined with DM.

The aim of this study is to investigate the association between RAR and all-cause mortality in critically ill patients with both CHD and DM within the ICU.

## 2 Methods

### 2.1 Data source

This study was a retrospective observational analysis. The data were obtained from the MIMIC-IV database (v 2.2). MIMIC-IV is a large, open-access database containing information on patients admitted to ICU at a large tertiary hospital in Boston from 2008 to 2019 (21). In order to access the database, the first author of this study, Sheng Chen, completed the Collaborative Institutional Training Initiative (CITI) course and passed both the “Conflicts of Interest” and “Data or Specimens Only Research” exams (ID: 12046100). The database was approved for research use by Massachusetts Institute of Technology and Beth Israel Deaconess Medical Center review boards, and informed consent was waived.

### 2.2 Inclusion and exclusion criteria

**Figure 1:**
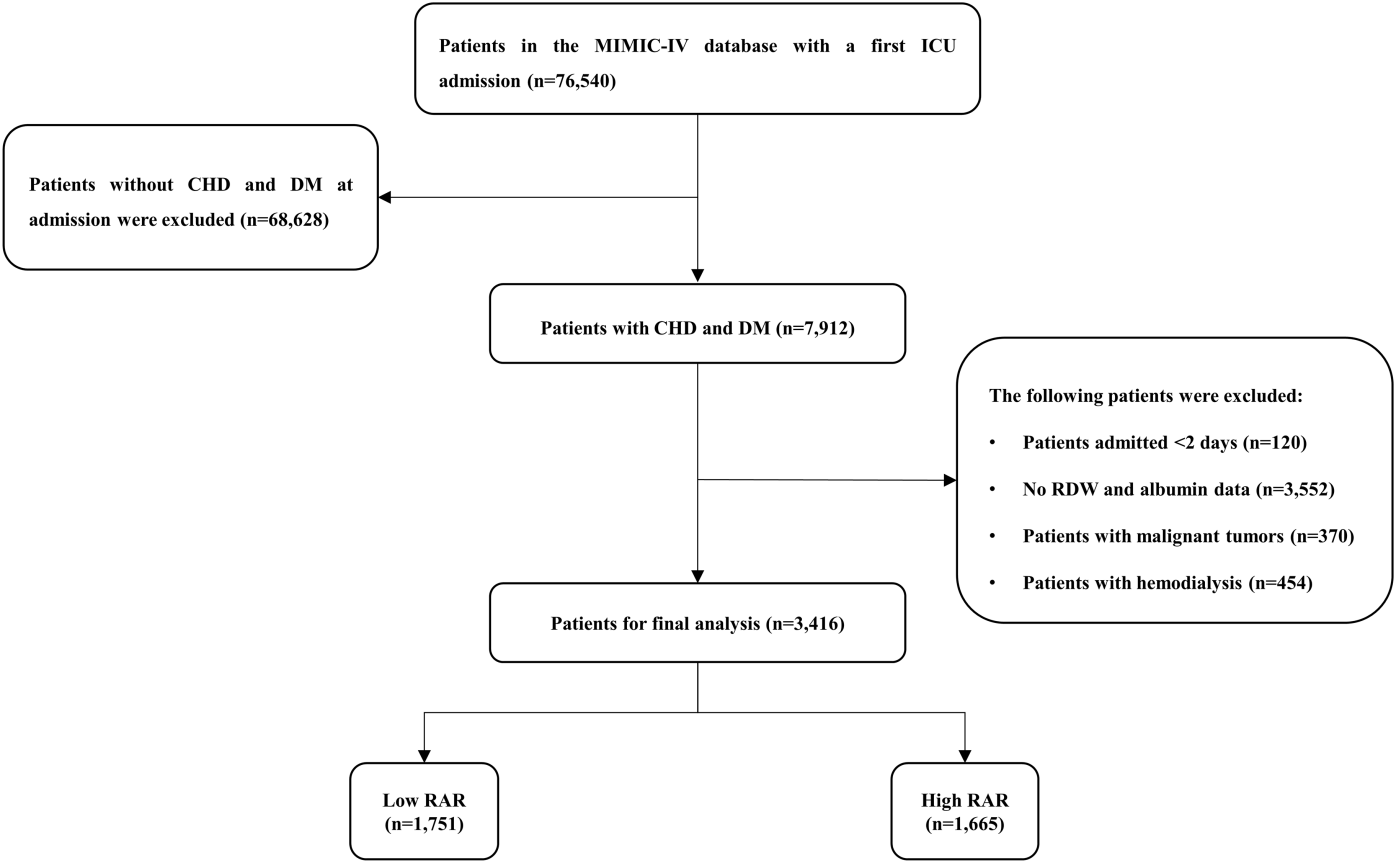
Study flow diagram in the present study. CHD, coronary heart disease; DM, diabetes mellitus; RAR, red blood cell distribution width-to-albumin ratio.

According to the International Classification of Diseases, Ninth Revision (ICD-9) and Tenth Revision (ICD-10) codes, adult patients (≥ 18 years) with CHD and DM who had been hospitalized in the ICU at first admission were included, and excluded acute coronary syndrome (ACS). Patients with the following criteria were excluded: (1) Patients admitted < 2 days; (2) No RDW and albumin data on the 24 hours of admission; (3) Patients with malignant tumors; (4) Patients with hemodialysis. Finally, a total of 3416 patients were included in the study cohort. We use the restricted cubic spline (RCS) model to determine the optimal cut-off of RAR as 4.26. The study population was divided into low RAR group (≤4.26) and high RAR group (> 4.26) according to the optimal cut-off (**Figure 1**).

### 2.3 Variables

We use the Structured Query Language (SQL) with PostgreSQL (version 15.2) to extract data. The baseline characteristics included age, gender, body mass index (BMI), and race. The vital signs included heart rate, systolic blood pressure (SBP), diastolic blood pressure (DBP), mean blood pressure (MBP), pulse oxygen saturation (SpO2), and temperature. The comorbidities were defined with ICD-9 or ICD-10 codes, including heart failure, atrial fibrillation, hypertension, hyperlipidemia, diabetes with complication, chronic obstructive pulmonary disease (COPD), chronic kidney disease (CKD) and stroke. The following laboratory variables within the first day after admission have also been extracted, including white blood cell (WBC), red blood cell (RBC), platelet, hemoglobin, hematocrit, RDW, albumin, potassium, sodium, calcium, chloride, Glycated hemoglobin (HbA1c), glucose, low density lipoprotein cholesterol (LDL-C), high density lipoprotein cholesterol (HDL-C), total cholesterol (TC), triglyceride (TG). The RAR was defined as the ratio of the RDW level to the albumin level. Furthermore, we also extracted the relevant severity scores, including Sequential Organ Failure Assessment (SOFA) score, Systemic inflammatory response syndrome (SIRS) score. Finally, we also extracted the clinical treatment, including antiplatelet drugs, statins, insulin and mechanical ventilation. The RAR was calculated as RDW (%)/albumin (g/dL).

To avoid bias, we excluded variables with more than 20% missing data, including BMI, calcium, HbA1c, LDL-C, HDL-C, TC, and TG. A single imputation method was used to impute missing values for variables with up to 5% missing. When dealing with variables that have missing values exceeding 5% but less than 20%, we employ the multiple imputation method to estimate and fill in the gaps, resulting in 5 new datasets. Subsequently, we selected the dataset with the highest Cronbach’s alpha to impute the missing values (**Supplementary Table 1**).

### 2.4 Study endpoints

The primary endpoint was 1-year mortality, defined as the all-cause mortality of patients within 1 year after admission to the ICU. And the secondary endpoint was 30-day mortality, 90-day mortality, hospital length of stay (LOS) and ICU LOS. The participants were followed from the date of admission to the date of death.

### 2.5 Statistical analysis

Student’s t-test or Kruskal-Wallis H test were used to compare continuous variables, which are presented as mean ± standard deviation or median with interquartile range. Categorical variables are presented as frequencies and percentages, and differences between groups were compared using Pearson chi-square test or Fisher’s exact test. We used RCS model to examine the associations between RAR and outcomes. Based on the above results, we determined the optimal cut-off value of RAR for all-cause mortality. We used Kaplan-Meier survival analysis to assess the incidence of endpoint events between groups according to different levels of RAR, and differences between groups were assessed using log-rank tests. The hazard ratio (HR) and 95% confidence interval (CI) between RAR and endpoints events were estimated using Cox proportional hazard models and adjusted for multiple models. We also calculated the variance inflation factor (VIF) to avoid overfitting the model due to multicollinearity between variables. Variables with VIF ≥ 5 were excluded. Based on the inclusion of two RAR groups, four multivariate models were constructed. Model 1: unadjusted; Model 2: adjusted for age, race, SBP and temperature; Model 3: adjusted for age, race, SBP, temperature, WBC, potassium, hypertension, hyperlipidemia, diabetes with complication, and stroke; Model 4: adjusted for age, race, SBP, temperature, WBC, potassium, hypertension, hyperlipidemia, diabetes with complication, stroke, antiplatelet drugs, statins, SOFA score, and SIRS score. To determine the consistency of the prognostic value of RAR for the primary outcomes, we further stratified the analyses by age (<65 and ≥65 years), gender, race (white, black and other), heart failure, atrial fibrillation, hypertension, hyperlipidemia, diabetes with complication, COPD, CKD, stroke, antiplatelet drugs, statins, insulin and mechanical ventilation. Likelihood ratio tests were used to examine interactions between the High RAR level and the variables used for stratification. The predictive ability of the RDW and RAR for hospital mortality was assessed using receiver operating characteristic (ROC) curves. All statistical analyses were conducted with SPSS 27.0 software (IBM SPSS Statistics, Armonk, NY, USA), R software version 4.2.2 (Institute for Statistics and Mathematics, Vienna, Austria) and GraphPad Prism version 9.3.0 (GraphPad software, San Diego, California, USA). Statistical significance was defined as a two-tailed P-value < 0.05.

## 3 Results

After reviewing the data for 76,540 patients admitted to the ICU from the MIMIC-IV database, a total of 3,416 patients were included in this study (**Figure 1**). There were 1208 (35.36%) females and 2208 (64.64%) males. The median age of the patients who were enrolled amounts to 71.41 years. The median RAR for all patients was 4.24. The 30-day mortality, 90-day mortality and 1-year mortality were 7.08%, 7.44% and 7.49%, respectively (**Table 1**).

**Table 1:** Baseline characteristics of Low RAR group and High RAR group.

Characteristics	Overall (n=3416)	Low RAR (≤ 4.26) (n=1751)	High RAR (> 4.26) (n=1665)	*P-value*
Age, years	71.41 (63.61-79.75)	69.95 (61.91-78.04)	73.22 (65.51-81.31)	< 0.001
Gender, n (%)				< 0.001
Female	1208 (35.36)	539 (30.78)	669 (40.18)	
Male	2208 (64.64)	1212 (69.22)	996 (59.82)	
Race, n (%)				< 0.001
White	2263 (66.25)	1127 (64.36)	1136 (68.23)	
Black	359 (10.51)	139 (7.94)	220 (13.21)	
Other	794 (23.24)	485 (27.70)	309 (18.56)	
Vital signs				
SBP, mmHg	115.65 (107.67-126.39)	115.61 (108.66-125.00)	115.73 (106.68-127.58)	0.289
DBP, mmHg	58.38 (52.57-65.08)	58.52 (53.16-64.56)	58.21 (51.95-65.53)	0.365
MBP, mmHg	74.96 (69.77-81.08)	75.39 (70.82-80.86)	74.26 (68.45-81.53)	< 0.001
Heart rate, times/min	81.75 (73.00-91.07)	81.04 (73.68-89.17)	82.38 (72.22-94.08)	< 0.001
SpO_2_, %	97.33 (96.09-98.54)	97.45 (96.26-98.54)	97.21 (95.90-98.55)	< 0.001
Temperature, °C	36.76 (36.54-37.01)	36.76 (36.54-36.99)	36.77 (36.52-37.05)	0.366
Comorbidities, n (%)				
Heart failure	2107 (61.68)	884 (50.48)	1223 (73.45)	< 0.001
Atrial fibrillation	1028 (30.09)	456 (26.04)	572 (34.35)	< 0.001
Hypertension	2234 (65.39)	1207 (68.93)	1027 (61.68)	< 0.001
Hyperlipidemia	2618 (76.64)	1393 (79.55)	1225 (73.57)	< 0.001
Diabetes with complication	1540 (45.08)	690 (39.41)	850 (51.05)	< 0.001
COPD	425 (12.44)	170 (9.71)	255 (15.32)	< 0.001
CKD	1615 (47.28)	655 (37.41)	960 (57.66)	< 0.001
Stroke	470 (13.76)	228 (13.02)	242 (14.53)	0.199
Laboratory parameters				
WBC, K/uL	9.10 (6.90-12.40)	8.50 (6.70-11.30)	10.00 (7.30-13.95)	< 0.001
RBC, K/uL	3.84 (3.31-4.38)	4.12 (3.66-4.55)	3.52 (3.05-4.01)	< 0.001
Platelet, K/uL	203.00 (156.00-257.00)	205.00 (165.00-248.00)	200.00 (146.50-266.00)	0.322
Hemoglobin, g/dL	11.30 (9.70-12.90)	12.30 (11.00-13.60)	10.20 (8.80-11.60)	< 0.001
Hematocrit, %	34.50 (29.90-38.80)	37.00 (33.30-40.60)	31.50 (27.50-35.80)	< 0.001
RDW, %	14.40 (13.40-15.70)	13.60 (13.00-14.40)	15.50 (14.40-17.10)	< 0.001
Albumin, g/dL	3.50 (3.00-3.90)	3.80 (3.60-4.10)	3.00 (2.60-3.30)	< 0.0001
Potassium, mEq/L	4.20 (3.90-4.60)	4.20 (3.90-4.50)	4.20 (3.80-4.70)	0.050
Sodium, mEq/L	139.00 (136.00-141.00)	139.00 (137.00-141.00)	138.32 (135.00-141.00)	0.005
Chloride, mEq/L	103.00 (99.00-106.00)	102.00 (100.00-105.00)	103.00 (99.00-107.00)	< 0.001
Glucose, mg/dL	147.66 (128.68-185.19)	142.33 (128.39-170.35)	156.63 (129.50-198.88)	< 0.001
RAR	4.24 (3.61-5.16)	3.63 (3.28-3.94)	5.18 (4.69-6.08)	< 0.0001
Scoring systems				
SOFA score	2.00 (1.00-4.00)	2.00 (1.00-4.00)	2.00 (0.00-3.00)	0.025
SIRS score	3.00 (2.00-3.00)	3.00 (2.00-3.00)	3.00 (2.00-3.00)	0.793
Clinical treatment, n (%)				
Use of antiplatelet drugs	2800 (81.97)	1549 (88.46)	1251 (75.14)	< 0.001
Use of statins	2766 (80.97)	1521 (86.86)	1245 (74.77)	< 0.001
Use of insulin	3225 (94.41)	1683 (96.12)	1542 (92.61)	< 0.001
Use of mechanical ventilation	1831 (53.60)	1065 (60.82)	766 (46.01)	< 0.001
Events				
30-day mortality, n (%)	242 (7.08)	64 (3.66)	178 (10.69)	< 0.001
90-day mortality, n (%)	254 (7.44)	65 (3.71)	189 (11.35)	< 0.001
1-year mortality, n (%)	256 (7.49)	66 (3.77)	190 (11.41)	< 0.001
LOS Hospital, days	9.08 (6.27-13.80)	8.44 (6.06-11.91)	10.08 (6.68-15.98)	< 0.001
LOS ICU, days	2.28 (1.29-4.18)	2.09 (1.23-3.36)	2.73 (1.45-5.15)	< 0.001

RAR, red blood cell distribution width-to-albumin ratio; SBP, systolic blood pressure; DBP, diastolic blood pressure; MBP, mean blood pressure; SpO2, pulse oxygen saturation; COPD, chronic obstructive pulmonary disease; CKD, chronic kidney disease; WBC, white blood cell; RBC, red blood cell; RDW, red blood cell distribution width; SOFA, Sequential Organ Failure Assessment; SIRS, Systemic inflammatory response syndrome; LOS, length of stay; ICU, intensive care unit.

### 3.1 Baseline characteristics

**Table 1** shows the baseline characteristics of the low RAR group and high RAR group. The median values of RAR of the two groups were 3.63 and 5.81, respectively (p < 0.0001). Patients with high RAR showed higher age, and had a faster heart rate, higher admission sickness scores, higher rates of heart failure, atrial fibrillation, diabetes with complication, COPD, CKD and stroke, higher WBC, RDW, glucose and lower MBP, SpO2, lower RBC, hemoglobin, hematocrit, platelets, albumin, lower proportion of antiplatelet drugs, statins, insulin compared with the low RAR group (p < 0.05). Compared with the low RAR group, the hospital LOS (8.44 days vs. 10.08 days, P < 0.001) and ICU LOS (2.09 days vs. 2.73 days, P < 0.001) of the high RAR group also increased.

In the 1-year mortality, the baseline characteristics of the survivor and non-survivor groups are summarized in **Table 2**. Patients in the non-survivor group were older and had a higher prevalence of heart failure and stroke (P < 0.05). Regarding of laboratory parameters, patients in the non-survivor group had higher levels of WBC, RDW, Potassium, and glucose, but lower levels of RBC, hemoglobin, hematocrit, and albumin (P < 0.05). SOFA scores and SIRS scores were higher in the non-survivor group than in the survivor group (P < 0.05). In clinical treatment, patients in the non-survivor group had lower proportion of antiplatelet drugs, statins and higher proportion of mechanical ventilation (p < 0.05). In the non-survivor group, the level of RAR was significantly higher than that of the survivor group (5.05 vs. 4.18, P < 0.001). More importantly, in comparison to the survivor group, the hospital LOS (9.07 days vs. 9.19 days, P = 0.042) and ICU LOS (2.22 days vs. 4.18 days, P < 0.001) also increased in the non-survivor group.

**Table 2:** Baseline characteristics of the Survivors group and Non-survivors group.

Characteristics	Overall (n=3416)	Survivors (n=3160)	Non-survivors (n=256)	*P-value*
Age, years	71.41 (63.61-79.75)	70.94 (63.22-79.25)	78.10 (70.38-85.17)	< 0.001
Gender, n (%)				0.731
Female	1208 (35.36)	1120 (35.44)	88 (34.37)	
Male	2208 (64.64)	2040 (64.56)	168 (65.63)	
Race, n (%)				0.036
White	2263 (66.25)	2098 (66.39)	165 (64.45)	
Black	359 (10.51)	341 (10.79)	18 (7.03)	
Other	794 (23.24)	721 (22.82)	73 (28.52)	
Vital signs				
SBP, mmHg	115.65 (107.67-126.39)	116.00 (107.9-126.74)	110.87 (103.95-121.69)	< 0.001
DBP, mmHg	58.38 (52.57-65.08)	58.48 (52.77-65.24)	57.51 (51.46-63.67)	0.014
MBP, mmHg	74.96 (69.77-81.08)	75.12 (69.98-81.26)	72.34 (66.69-78.87)	< 0.001
Heart rate, times/min	81.75 (73.00-91.07)	81.69 (73.16-90.77)	83.14 (72.22-96.11)	0.030
SpO_2_, %	97.33 (96.09-98.54)	97.33 (96.09-98.53)	97.42 (95.80-98.68)	0.925
Temperature, °C	36.76 (36.54-37.01)	36.76 (36.54-37.01)	36.72 (36.38-37.12)	0.134
Comorbidities, n (%)				
Heart failure	2107 (61.68)	1930 (61.07)	177 (69.14)	0.011
Atrial fibrillation	1028 (30.09)	942 (29.81)	86 (33.59)	0.204
Hypertension	2234 (65.39)	2104 (66.58)	130 (50.78)	< 0.001
Hyperlipidemia	2618 (76.64)	2471 (78.19)	147 (57.42)	< 0.001
Diabetes with complication	1540 (45.08)	1438 (45.51)	102 (39.84)	0.080
COPD	425 (12.44)	395 (12.50)	30 (11.72)	0.716
CKD	1615 (47.28)	1480 (46.84)	135 (52.73)	0.069
Stroke	470 (13.76)	409 (12.94)	61 (23.83)	< 0.001
Laboratory parameters				
WBC, K/uL	9.10 (6.90-12.40)	9.00 (6.90-12.20)	11.20 (8.00-15.43)	< 0.001
RBC, K/uL	3.84 (3.31-4.38)	3.85 (3.33-4.39)	3.57 (3.07-4.12)	< 0.001
Platelet, K/uL	203.00 (156.00-257.00)	203.50 (157.00-256.00)	201.50 (151.00-269.75)	0.936
Hemoglobin, g/dL	11.30 (9.70-12.90)	11.40 (9.80-12.97)	10.60 (9.00-12.07)	< 0.001
Hematocrit, %	34.50 (29.90-38.80)	34.60 (30.00-38.90)	32.80 (27.85-38.28)	< 0.001
RDW, %	14.40 (13.40-15.70)	14.30 (13.40-15.60)	15.40 (14.10-17.08)	< 0.001
Albumin, g/dL	3.50 (3.00-3.90)	3.50 (3.00-3.90)	3.10 (2.60-3.60)	< 0.001
Potassium, mEq/L	4.20 (3.90-4.60)	4.20 (3.90-4.60)	4.40 (3.93-5.00)	< 0.001
Sodium, mEq/L	139.00 (136.00-141.00)	139.00 (136.00-141.00)	138.00 (135.00-141.00)	0.101
Chloride, mEq/L	103.00 (99.00-106.00)	103.00 (99.00-106.00)	102.00 (98.00-107.00)	0.282
Glucose, mg/dL	147.66 (128.68-185.19)	146.46 (128.38-181.38)	171.68 (138.56-215.02)	< 0.001
RAR	4.24 (3.61-5.16)	4.18 (3.57-5.09)	5.07 (4.25-6.38)	< 0.001
Scoring systems				
SOFA score	2.00 (1.00-4.00)	2.00 (0.00-4.00)	3.00 (1.00-5.00)	< 0.001
SIRS score	3.00 (2.00-3.00)	3.00 (2.00-3.00)	3.00 (2.00-3.00)	< 0.001
Clinical treatment, n (%)				
Use of antiplatelet drugs	2800 (81.97)	2620 (82.91)	180 (70.31)	< 0.001
Use of statins	2766 (80.97)	2592 (82.03)	174 (67.97)	< 0.001
Use of insulin	3225 (94.41)	2982 (94.37)	243 (94.92)	0.710
Use of mechanical ventilation	1831 (53.60)	1672 (52.91)	159 (62.11)	0.005
Events				
LOS Hospital, days	9.08 (6.27-13.80)	9.07 (6.39-13.79)	9.19 (4.92-14.57)	0.042
LOS ICU, days	2.28 (1.29-4.18)	2.22 (1.28-4.04)	4.18 (2.06-7.88)	< 0.001

SBP, systolic blood pressure; DBP, diastolic blood pressure; MBP, mean blood pressure; SpO2, pulse oxygen saturation; COPD, chronic obstructive pulmonary disease; CKD, chronic kidney disease; WBC, white blood cell; RBC, red blood cell; RDW, red blood cell distribution width; SOFA, Sequential Organ Failure Assessment; SIRS, Systemic inflammatory response syndrome; LOS, length of stay; ICU, intensive care unit.

### 3.2 RAR level and hospital mortality

**Figure 2:**
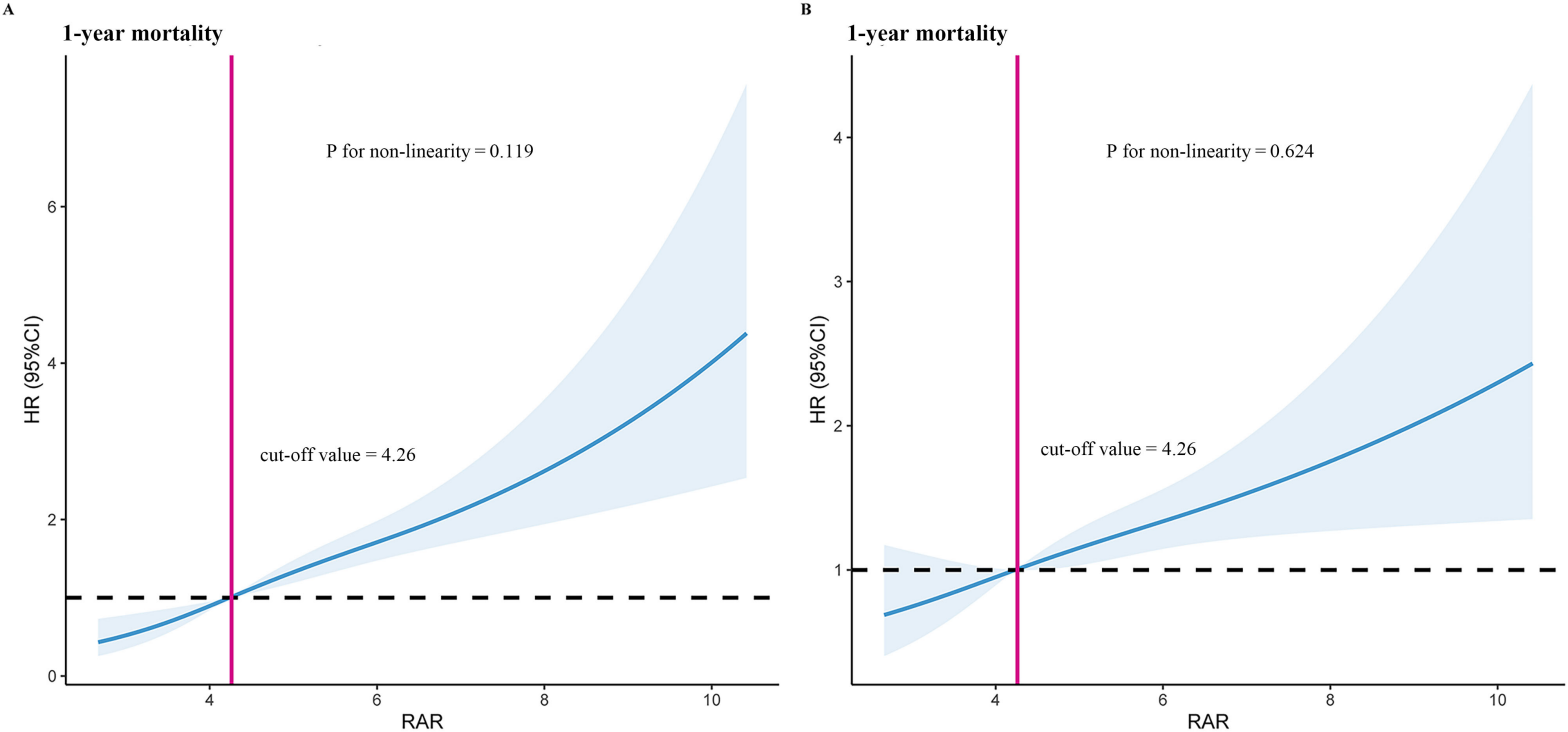
Restricted cubic spline model for 1-year mortality. **(A)** Unadjusted restricted cubic spline model for 1-year mortality. **(B)** Fully adjusted restricted cubic spline model for 1-year mortality. Adjusted risk factors including age, race, SBP, temperature, WBC, potassium, hypertension, hyperlipidemia, diabetes with complication, stroke, antiplatelet drugs, statins, SOFA score, and SIRS score. HR, hazard ratio; RAR, red blood cell distribution width-to-albumin ratio.

Using RCS model, we found that the relationship between RAR level and 1-year mortality was linear (P for non-linearity = 0.119 and P for non-linearity = 0.624, respectively) (**Figure 2**). Based on the RCS model, we have concluded that the optimal cut-off value for RAR is 4.26. Moreover, after undergoing complete adjustment, the optimal cut-off value for RAR remains unchanged at 4.26.

**Figure 3:**
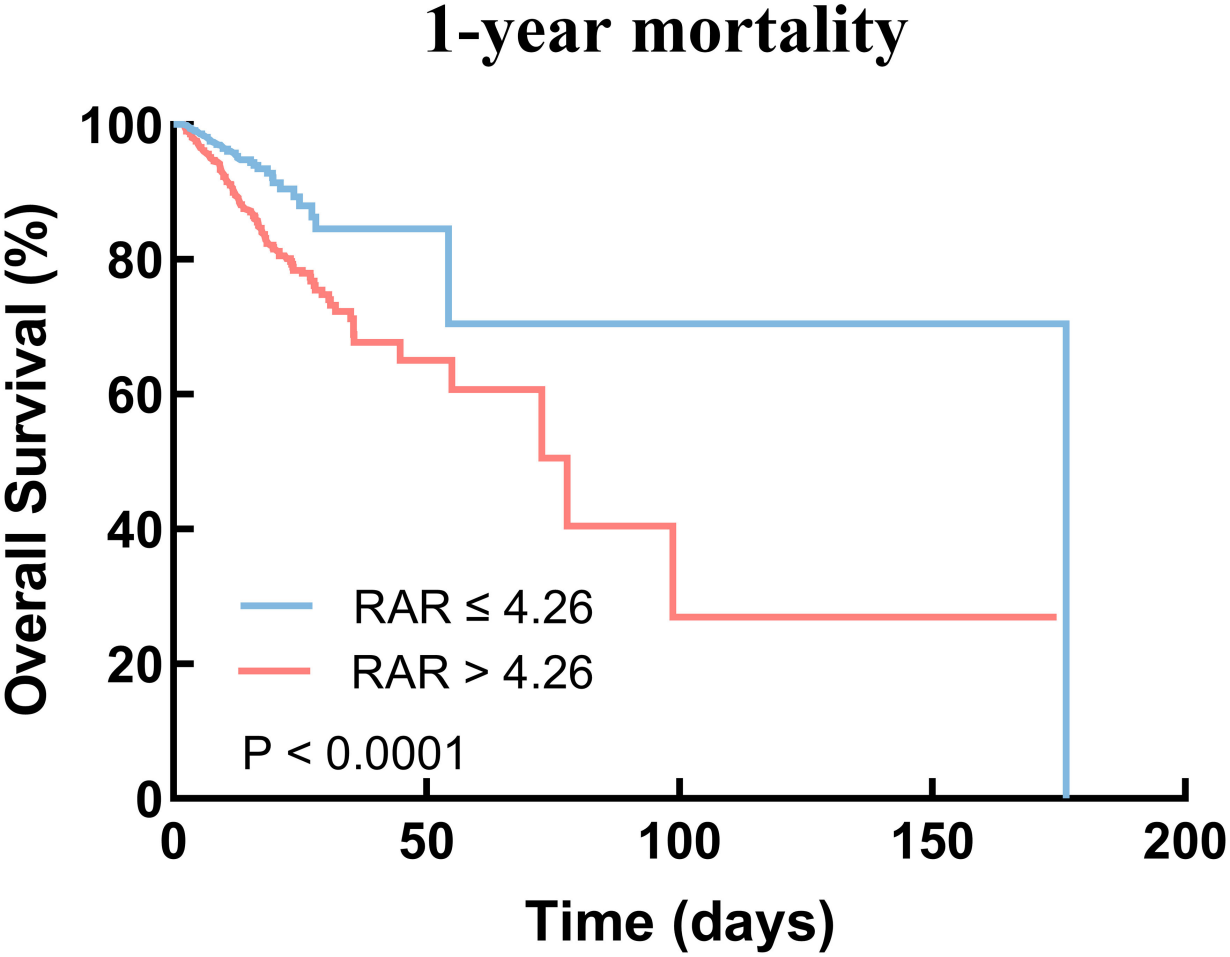
Kaplan–Meier survival analysis curves for 1-year mortality. RAR, red blood cell distribution width-to-albumin ratio.

We plotted Kaplan-Meier survival analysis curves to observe the incidence of the endpoint events between low RAR group and high RAR group, as shown in **Figure 3**. In the 1-year mortality, a statistically significant difference in mortality was observed between the two groups (log-rank P < 0.0001, **Figure 3**). Significance was also observed in the 30-day mortality (log-rank P < 0.0001, see **Supplementary Figure 1A**) and 90-day mortality (log-rank P < 0.0001, see **Supplementary Figure 1B**).

Firstly, we employed univariate Cox regression analysis to identify variables with statistical significance (P < 0.1) (**Supplementary Table 2**). Secondly, we excluded variables with a VIF ≥ 5 based on collinearity diagnostics (**Supplementary Table 3**). The remaining variables were used to adjust the multivariate Cox regression analysis model.

We used multivariate Cox regression analysis model, with the low RAR group as the reference group, to determine the association between the RAR and hospital mortality. The results demonstrated that it was significant associated with 1-year mortality in both unadjusted model 1 (low RAR vs. high RAR: HR, 2.25 [95% CI 1.69–2.98] P < 0.001) and fully adjusted model 4 (low RAR vs. high RAR: HR, 1.58 [95% CI 1.17–2.13] P = 0.003) (**Table 3**). Further, the multivariate Cox regression analysis model for 30-day and 90-day mortality showed simiRAR results (**Table 3**).

**Table 3:** Cox proportional hazard ratios (HR) for all-cause mortality.

Categories	Model 1 HR (95% CI)	P value	Model 2 HR (95% CI)	P value	Model 3 HR (95% CI)	P value	Model 4 HR (95% CI)	P value
Primary endpoint								
1-year mortality								
RAR	1.32 (1.23-1.42)	< 0.001	1.28 (1.19-1.38)	< 0.001	1.23 (1.13-2.33)	< 0.001	1.18 (1.09-1.28)	< 0.001
Low RAR (≤ 4.26)	Ref.		Ref.		Ref.		Ref.	
High RAR (> 4.26)	2.25 (1.69-2.98)	< 0.001	2.05 (1.53-2.73)	< 0.001	1.75 (1.29-2.35)	< 0.001	1.58 (1.17-2.13)	0.003
Secondary endpoint								
30-day mortality								
RAR	1.32 (1.23-1.42)	< 0.001	1.28 (1.19-1.38)	< 0.001	1.22 (1.13-1.32)	< 0.001	1.17 (1.08-1.27)	< 0.001
Low RAR (≤ 4.26)	Ref.		Ref.		Ref.		Ref.	
High RAR (> 4.26)	2.19 (1.65-2.93)	< 0.001	2.02 (1.51-2.70)	< 0.001	1.71 (1.27-2.31)	< 0.001	1.53 (1.17-2.07)	0.006
90-day mortality								
RAR	1.32 (1.23-1.42)	< 0.001	1.28 (1.19-1.38)	< 0.001	1.23 (1.13-1.33)	< 0.001	1.18 (1.09-1.28)	< 0.001
Low RAR (≤ 4.26)	Ref.		Ref.		Ref.		Ref.	
High RAR (> 4.26)	2.24 (1.68-2.98)	< 0.001	2.04 (1.53-2.73)	< 0.001	1.74 (1.29-2.34)	< 0.001	1.58 (1.17-2.13)	0.003

HR, hazard ratio; CI, confidence interval; Ref., reference; RAR, red blood cell distribution width-to-albumin ratio.

Model 1: unadjusted.

Model 2: adjusted for age, race, SBP and temperature.

Model 3: adjusted for age, race, SBP, temperature, WBC, potassium, hypertension, hyperlipidemia, diabetes with complication, and stroke.

Model 4: adjusted for age, race, SBP, temperature, WBC, potassium, hypertension, hyperlipidemia, diabetes with complication, stroke, antiplatelet drugs, statins, SOFA score, and SIRS score.

### 3.3 Subgroup analysis

**Figure 4:**
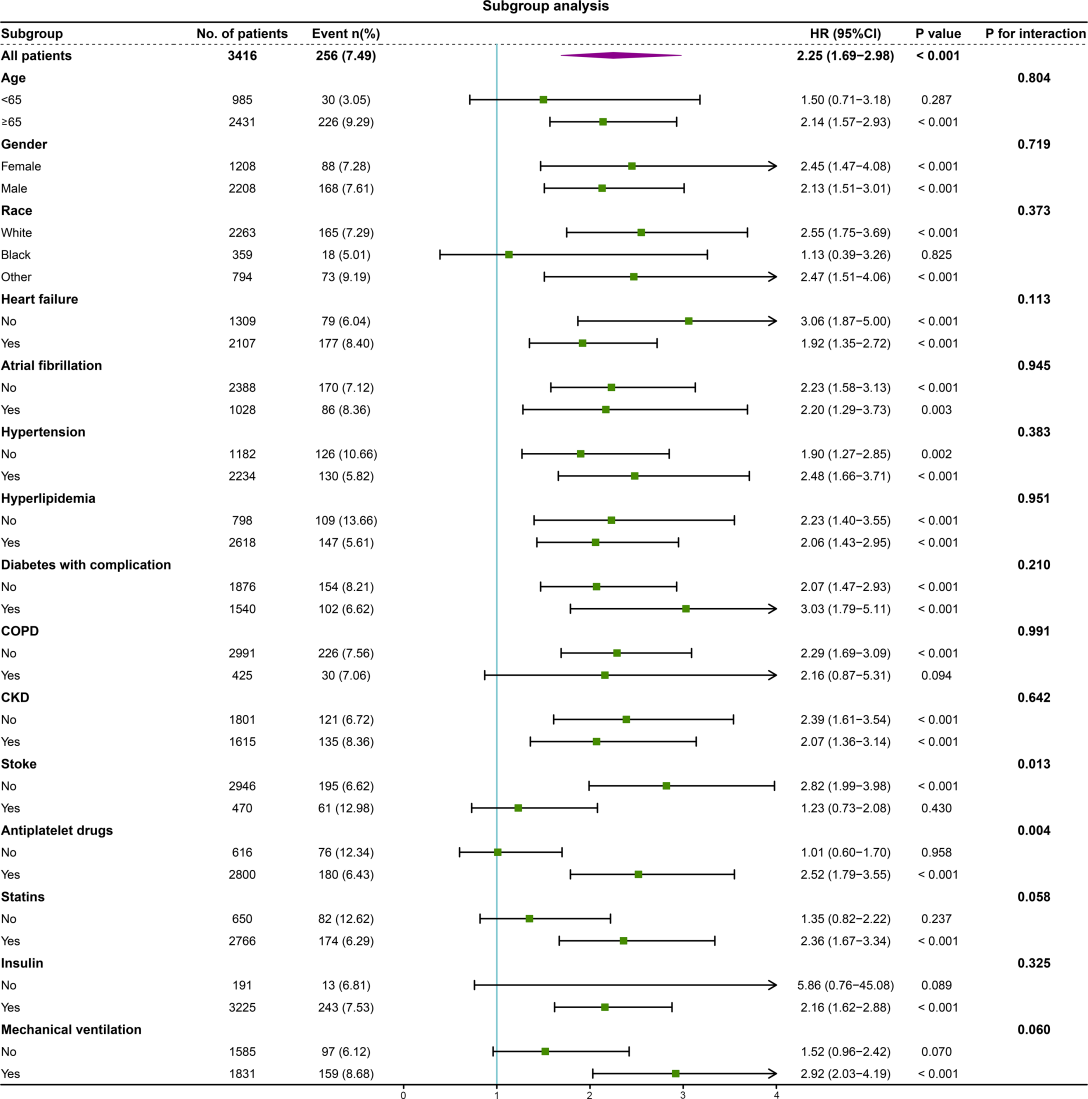
Forest plot of the relationship between 1-year mortality and high RAR for subgroup analysis. COPD, chronic obstructive pulmonary disease; CKD, chronic kidney disease.

We performed subgroup analysis to assess the association between the high RAR and 1-year mortality, including age, gender, race, heart failure, atrial fibrillation, hypertension, hyperlipidemia, diabetes with complication, COPD, CKD, stroke, antiplatelet drugs, statins, insulin and mechanical ventilation (**Figure 4**). Interestingly, the predictive value of high RAR appears to be more significant in patients administered with antiplatelet drugs [HR (95% CI) antiplatelet drugs 2.52 (1.79-3.55) vs. no antiplatelet drugs 1.01 (0.60-1.70), P for interaction = 0.004]. Moreover, the same phenomenon is also observed in non-stroke patients [HR (95% CI) no stroke 2.82 (1.99-3.98) vs. stroke 1.23 (0.73-2.08), P for interaction = 0.013]. Significant interaction was not observed in other subgroups.

### 3.4 ROC curve analysis and prediction of mortality

**Figure 5:**
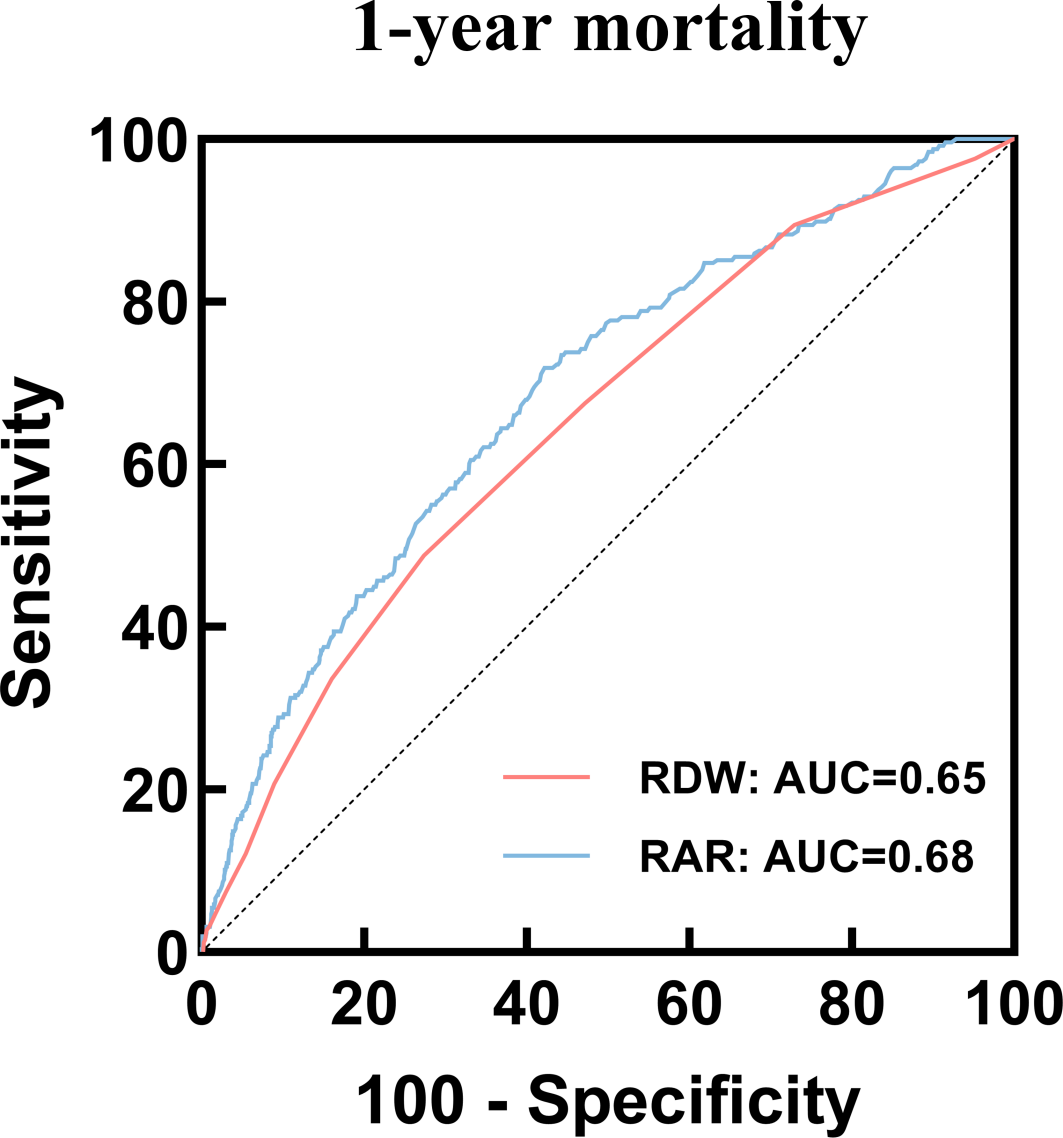
ROC curves of RAR and RDW to predict 1-year mortality. ROC, receiver operating characteristic; RAR, Red blood cell distribution width-to-albumin ratio; RDW, Red blood cell distribution width.

We plotted ROC curves of RDW and RAR to predict hospital all-cause mortality. In the 1-year mortality, the result showed that the area under the curve (AUC) of RAR [0.68 (95% CI: 0.65-0.72)] was superior to that of RDW [0.65 (95% CI: 0.61-0.68)] (**Figure 5**), and the AUC difference between the RAR and RDW was statistically significant (P = 0.015) (**Supplementary Table 4**). Further, the ROC curve analysis for 30-day and 90-day mortality showed similar results (**Supplementary Figure 2, Supplementary Table 4**).

## 4 Discussion

Critically ill patients in the ICU experience heightened mortality rates, particularly when presenting with both CHD and DM. Early and accurate assessment is pivotal in stratifying disease severity and determining optimal treatment strategies to improve patient prognosis. Our research findings establish a significant correlation between increasing RAR and elevated hospital mortality rates. Furthermore, whether analyzed as a continuous or categorical variable, RAR exhibits a positive association with hospital mortality among critically ill patients with CHD and DM in the ICU. Moreover, our RCS model identifies a critical threshold of 4.26 for predicting mortality risk.

It is worth noting that DM commonly coexists with CHD, with these conditions mutually influencing one another. Inflammation and oxidative stress are recognized as key contributors to the development of both CHD and DM (22–24). The development of CHD is associated with atherosclerosis, wherein inflammation is known to contribute to its pathogenesis (25). Additionally, patients with DM often exhibit chronically elevated inflammation levels (26). DM constitutes a significant risk factor for accelerated atherosclerosis, thereby exacerbating the progression of CHD. Notably, individuals with DM and CHD face an increased mortality risk ranging from two to four times higher than those without DM (27).

In the past, RDW was considered a hematology index that reflected the volume of RBC. However, more and more studies have shown that both inflammation and oxidative stress can result in an elevation of RDW levels (28, 29). Furthermore, an increasing number of studies suggest that RDW may function as an indicator of inflammation (7, 28). Notably, a study revealed that patients with high RDW values also exhibited elevated levels of high-sensitivity C-reactive protein (hs-CRP) (28). Hs-CRP is a validated marker for cardiovascular disease (CVD) and holds predictive value for future CVD events (30). These evidences underscore the potential of RDW as a prognostic indicator for CVD.

More recent investigations have increasingly recognized the association between elevated RDW levels and CVD mortality (31, 32). Furthermore, RDW has been linked to negative prognoses among patients with DM (33). A cohort study involving 233 patients demonstrated an elevation in RDW levels within the population affected by both coronary artery disease and DM (34). Additionally, higher RDW values have been correlated with an augmented risk of CVD in patients with DM (35). Notably, a significant correlation exists between RDW and the presence and severity of coronary artery calcification (36), with DM patients exhibiting high coronary artery calcification scores displaying elevated RDW levels (37). Some researchers propose a notable positive correlation between RDW and glycated hemoglobin (HbA1c) (38), suggesting that chronic hyperglycemia may mediate the relationship between elevated RDW levels and the progression of CVD (39). Consequently, elevated RDW levels may serve as an indicator of unfavorable prognosis among patients with both CHD and DM. However, it is important to acknowledge that as mentioned in the introduction, RDW levels can be influenced by other factors, and relying solely on RDW level predictions may not yield reliable results.

Albumin, a multifunctional protein involved in antioxidant defense, anti-inflammatory processes, and the maintenance of vascular endothelial function (40), plays a crucial role in mitigating the detrimental effects of inflammation on the body (12, 40). These essential functions contribute to inhibiting the development of coronary atherosclerosis, thereby influencing the onset and progression of CHD. Notably, low levels of albumin can intensify inflammatory responses, while inflammation can accelerate the advancement of atherosclerosis (14). Previous studies have demonstrated that reduced albumin levels are associated with an increased risk of CVD events (13, 14). Additionally, research has revealed a negative correlation between serum albumin levels and the risk of developing CHD (41). It is worth noting that serum albumin can be influenced by various factors such as chronic diseases, malnutrition, and inflammation.

The RAR serves as a composite indicator that combines both nutritional and inflammatory status, exhibiting a stronger correlation with mortality compared to single indicators. This characteristic renders it a valuable prognostic biomarker for various diseases. Studies have consistently shown that elevated RAR levels are associated with all-cause mortality in multiple cardiovascular diseases, including atrial fibrillation, heart failure, and aortic aneurysm (42–44). Li et al. demonstrated that high RAR levels are indicative of poor prognosis in patients with acute myocardial infarction (16). Moreover, RAR exhibits excellent predictive capability for all-cause mortality in patients undergoing percutaneous coronary intervention (PCI), surpassing the use of RDW or albumin alone (45). Recent investigations have further revealed the relationship between RAR and prognosis in patients with DM. Specifically, elevated RAR levels are linked to unfavorable outcomes in DM-related complications (17, 19). Huang et al. provided evidence that RAR levels in patients with CHD are associated with carotid plaque formation, with a particularly robust relationship observed between carotid plaque and RAR in patients affected by both CHD and DM (20). To the best of our knowledge, no previous research has explored the correlation between RAR and prognosis specifically in critically ill patients with coexisting CHD and DM. Therefore, our study represents the first to underscore the significance of RAR as a prognostic indicator in this patient population.

The prognosis for critically ill patients with coexisting CHD and DM is generally unfavorable. Our study reveals a significant correlation between elevated RAR levels and an increased risk of all-cause mortality in this patient population. Specifically, we observed that higher RAR levels were associated with elevated 30-day, 90-day, and 1-year mortality. One study showed that increased stress hyperglycemia ratio at hospital admission in stroke patients are associated with increased in-hospital mortality and length of stay (46). Consistent with the above result, our study also showed that the high RAR group exhibited prolonged hospital LOS and ICU LOS. This may explain that RAR happens to be associated with elevated blood glucose, suggesting poor prognosis in patients, and can provide a valuable basis for our findings. This undoubtedly places a substantial burden on both families and society. Hence, it is imperative to prioritize the prognosis of critically ill patients and implement early identification strategies to improve their healthcare outcomes. Previously, Li et al. demonstrated that high RAR levels were linked to increased 90-day mortality in patients with acute myocardial infarction (16). Similarly, in a retrospective study involving 707 post-PCI patients, Weng et al. identified a positive association between RAR and adverse outcomes (45). In line with these previous findings, our study reveals that elevated RAR levels are associated with higher mortality rates in our patient cohort. We conducted multivariate Cox proportional risk analysis and Kaplan-Meier survival analysis, which consistently demonstrated this relationship. Notably, these results remained significant even after adjusting for potential confounding factors.

Similarly, the RCS model demonstrated a significant linear correlation between RAR and 1-year mortality in critically ill patients with both CHD and DM. Importantly, our research findings indicate that the cutoff value of RAR for predicting 1-year mortality is 4.26. When the RAR level exceeds 4.26, the risk of death in these patients is significantly elevated, surpassing twice that of the low RAR group. Moreover, this relationship persists even after adjusting for multiple confounding factors. In a study conducted by Li et al. involving 826 patients with acute myocardial infarction, individuals with RAR levels above 4.0 ml/g demonstrated a higher risk of all-cause mortality (16). Although slightly lower than the findings of our research, it is important to note that our study excluded individuals with ACS, including acute myocardial infarction. Mainly due to the rapid changes in the condition of patients with ACS, there are many influencing factors that may affect the predicted results. At the same time, RAR can be used to predict the progress of inflammation, which may be more suitable for predicting the prognosis of patients with CHD and DM. Therefore, our results provide valuable insights for the early identification of patients at high risk of mortality, and monitoring RAR could aid in the improved management of these individuals. This is particularly vital for enhancing clinical care and reducing future adverse events.

Furthermore, our subgroup analysis revealed consistent predictive values of RAR across various demographic factors, including age, gender, race, and most comorbidities. However, we observed that high RAR levels had a more significant predictive value in patients receiving antiplatelet therapy. It is important to note that patients with CHD, especially those with CHD and DM, typically receive standardized secondary prevention treatment (47, 48). In our study cohort, the utilization rate of antiplatelet drugs exceeded 80%. Hence, our research findings can be effectively applied to a large proportion of CHD patients, particularly those with coexisting DM. However, we also found that RAR demonstrated more effectiveness in predicting mortality risk among patients without a history of stroke. This observation may be attributed to the fact that patients diagnosed with stroke tend to undergo more rigorous treatment regimens, which could potentially impact the prognostic significance of RAR in this particular subgroup.

Finally, our study provides compelling evidence that RAR serves as a superior predictor of mortality in critically ill patients with both CHD and DM. The results of the ROC analysis reveal that the AUC for RAR in predicting 30-day, 90-day, and 1-year mortality is 0.68, which is significantly higher than that of RDW (AUC: 0.65). These findings align with previous research (45), highlighting that the combination of RDW and albumin levels may exhibit a stronger correlation with mortality when employed together, particularly among critically ill patients presenting with both CHD and DM.

In assessing the inflammatory response, RAR may be a more effective tool compared to other single identification biomarkers. Additionally, as it reflects the inflammatory response, it can serve as a prognostic marker and thus help identify high-risk patients. The rapid and easy acquisition of RAR through laboratory testing allows for routine screening in clinical practice. Especially for ICU patients, particularly those with CHD and DM, given the complexity and critical nature of their condition, monitoring RAR routinely is advisable. Likewise, even prior to admission to the ICU, RAR can serve as a simple yet relatively reliable indicator for stratifying high-risk patients with CHD and DM. However, when using RAR, its shortcomings should also be emphasized, such as being affected by nutritional status, liver disease, and certain medications.

The strength of our study lies in confirming a significant association between elevated RAR and increased hospital mortality among critically ill patients with both CHD and DM. Moreover, RAR serves as a straightforward and easily accessible indicator compared to alternative measures. However, it is essential to acknowledge several limitations of our study. Firstly, being a single-center retrospective study, it lacks the ability to establish causality. Secondly, despite controlling for confounding variables through subgroup analysis, there may still exist unadjusted confounders, such as smoking status, body mass index (BMI), glycated hemoglobin (HbA1c), blood lipid levels, liver diseases and medications, which could have influenced our findings. Finally, the database used in our study does not provide corresponding data on the causes of hospitalization and death, making it impossible to assess disease progression accurately. Therefore, future prospective studies should aim to improve the inclusion of corresponding imaging examinations, such as coronary artery angiography and echocardiography, when measuring RAR. This will allow for the evaluation of hospitalization causes and the assessment of disease progression in critically ill patients with both CHD and DM.

## 5 Conclusions

Based on our research findings, a positive correlation between RAR and hospital mortality in critically ill patients with both CHD and DM has been observed. Our study suggests that elevated RAR levels have the potential to serve as a predictive indicator for hospital mortality, thus aiding in risk stratification and prognosis prediction in this patient population. Furthermore, these findings hold promise for practical application among a substantial number of patients with CHD and DM who have received secondary prevention treatment for CHD. It is recommended that further prospective studies be conducted to validate the predictive efficacy of RAR in critically ill patients with coexisting CHD and DM.

## Data Availability

The data analyzed in this study was obtained from the Medical Information Mart for Intensive Care IV (MIMIC-IV) database, the following licenses/restrictions apply: To access the files, users must be credentialed users, complete the required training (CITI Data or Specimens Only Research) and sign the data use agreement for the project. Requests to access these datasets should be directed to PhysioNet, https://physionet.org/, DOI: 10.13026/6mm1-ek67.
