# Supplementary material for: Prognostic value of red blood cell distribution width-to-albumin ratio in ICU patients with coronary heart disease and diabetes mellitus
Source: Front Endocrinol (Lausanne). 2024 Sep 25;15:1359345. doi: 10.3389/fendo.2024.1359345 (PMC11461254; doi:10.3389/fendo.2024.1359345)
Supplement: Supplementary file 1 [file DataSheet1.docx]

Supplementary Material

Prognostic value of red blood cell distribution width-to-albumin ratio in ICU patients with coronary heart disease and diabetes mellitus

**Sheng Chen^1^, Senhong Guan^1^, Zhaohan Yan^1^, Fengshan Ouyang^2^, Shuhuan Li^3^, Lanyuan Liu^4^, Liuer Zuo^5*^, Yuli Huang^1*^, Jiankai Zhong^1*^**

*** Correspondence:** Jiankai Zhong: doctor-zh@smu.edu.cn; Yuli Huang: hyuli821@smu.edu.cn; Liuer Zuo: 13500276597@163.com

* These authors are co-corresponding authors.

# Supplementary Figures and Tables

## Supplementary Figures


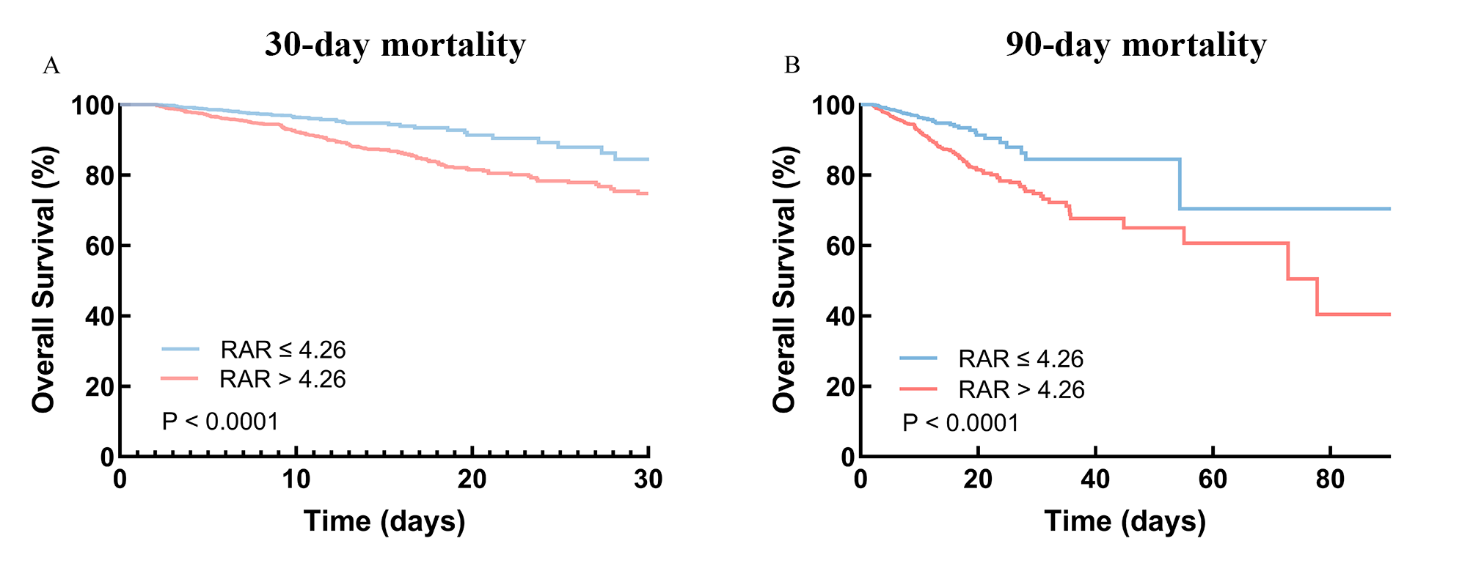


**Supplementary Figure 1.** Kaplan-Meier survival analysis curves for hospital mortality. (A) Kaplan-Meier survival analysis curves for 30-day mortality. (B) Kaplan-Meier survival analysis curves for 90-day mortality. Abbreviations: RAR, Red blood cell distribution width-to-albumin ratio


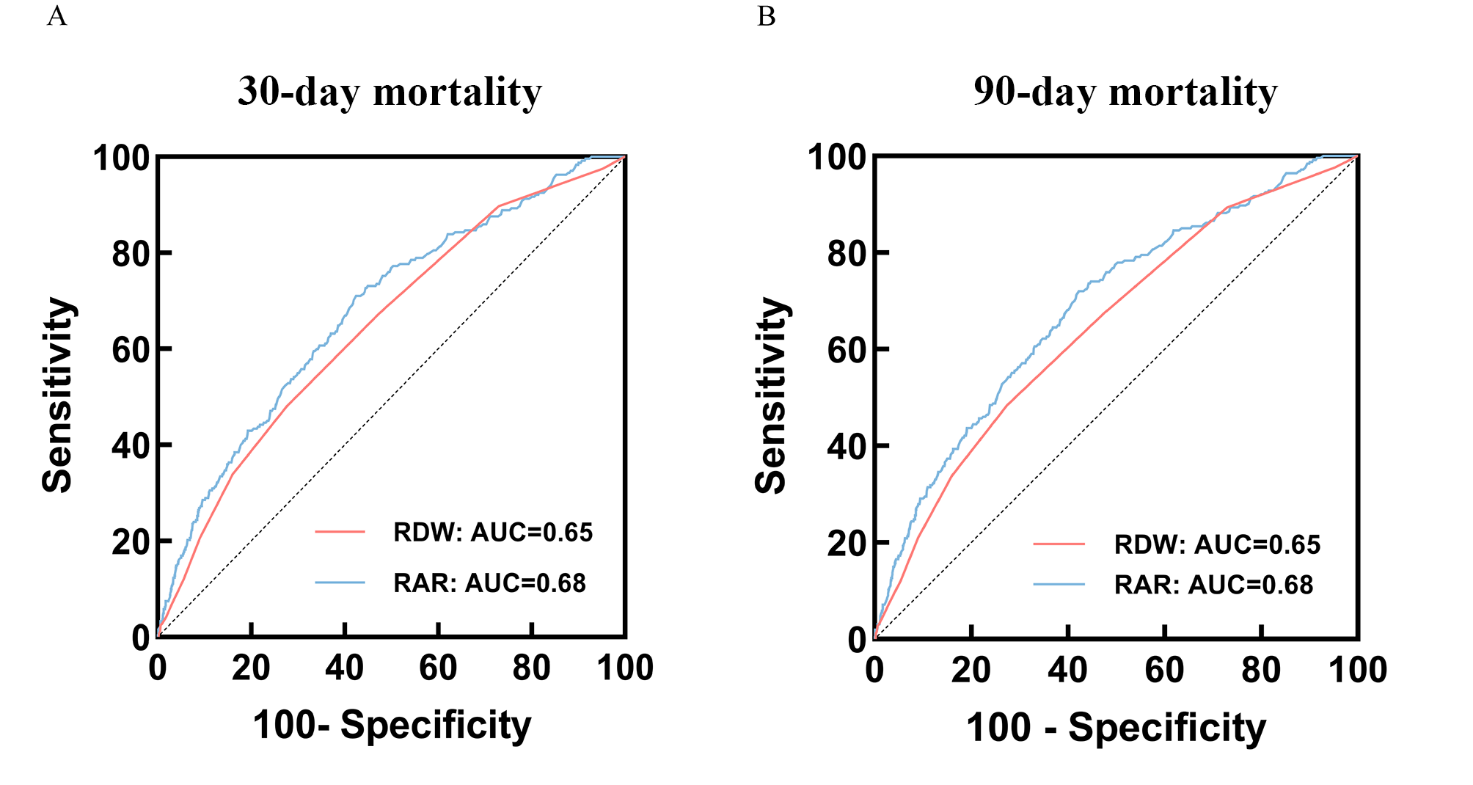


**Supplementary Figure 2.** ROC curve analysis and prediction of mortality. (A) ROC curves of RAR and RDW to predict 30-day mortality. (B) ROC curves of RAR and RDW to predict 90-day mortality. Abbreviations: ROC, Receiver operating characteristic; RAR, Red blood cell distribution width-to-albumin ratio; RDW, Red blood cell distribution width

## Supplementary Table

**Supplementary Table 1. Missing number for all variables**

| Variables | Missing number (%) |
| --- | --- |
| Age | 0 (0) |
| Gender | 0 (0) |
| BMI | 1218 (35.65%) |
| Heart rate | 6 (00.17%) |
| SBP | 7 (00.20%) |
| DBP | 7 (00.20%) |
| MBP | 7 (00.20%) |
| temperature | 218 (6.38%) |
| SpO_2_ | 8 (00.23%) |
| Heart failure | 0 (0) |
| Atrial fibrillation | 0 (0) |
| Hypertension | 0 (0) |
| Hyperlipidemia | 0 (0) |
| Diabetes with complication | 0 (0) |
| COPD | 0 (0) |
| CKD | 0 (0) |
| Stroke | 0 (0) |
| WBC | 0 (0) |
| RBC | 0 (0) |
| Platelet | 6 (00.17%) |
| Hemoglobin | 1 (00.02%) |
| Hematocrit | 0 (0) |
| RDW | 0 (0) |
| Albumin | 0 (0) |
| Potassium | 63 (1.84%) |
| Sodium | 64 (1.87%) |
| Calcium | 831 (24.33%) |
| Chloride | 20 (00.58%) |
| HbA1c | 1788 (52.34%) |
| Glucose | 7 (00.20%) |
| LDL-C | 2915 (85.33%) |
| HDL-C | 2893 (84.69%) |
| TG | 2698 (78.98%) |
| TC | 2847 (83.34%) |
| RAR | 0 (0) |
| SOFA score | 0 (0) |
| SIRS score | 0 (0) |
| Antiplatelet drugs | 0 (0) |
| Statins | 0 (0) |
| Insulin | 0 (0) |
| Ventilator use | 0 (0) |

BMI, body mass index; SBP, systolic blood pressure; DBP, diastolic blood pressure; MBP, mean blood pressure; SpO2, pulse oxygen saturation; COPD, chronic obstructive pulmonary disease; CKD, chronic kidney disease; WBC, white blood cell; RBC, red blood cell; RDW, red blood cell distribution width; HbA1c, glycosylated hemoglobin; LDL-C, low density lipoprotein cholesterol; HDL-C, high density lipoprotein cholesterol; TC, total cholesterol; TG, triglyceride; RAR, RDW-albumin ratio; SOFA, Sequential Organ Failure Assessment; SIRS, Systemic inflammatory response syndrome.

**Supplementary Table 2. Univariate Cox regression analysis for 1-year mortality.**

| Characteristic | HR (95%CI) | P-value |
| --- | --- | --- |
| Male (vs. Female) | 1.05 (0.81-1.37) | 0.690 |
| Age | 1.06 (1.04-1.07) | < 0.001 |
| Race |  |  |
| White (vs. Other)  Black (vs. Other)  Heart rate | 0.74 (0.56-0.97)  0.45 (0.27-0.76)  1.00 (0.99-1.01) | 0.033  0.003  0.386 |
| SBP | 0.98 (0.97-0.99) | < 0.001 |
| DBP | 0.98 (0.97-0.99) | 0.003 |
| MBP | 0.97 (0.96-0.98) | < 0.001 |
| SpO_2_ | 0.96 (0.90-1.02) | 0.181 |
| Temperature | 0.56 (0.46-0.67) | < 0.001 |
| Heart failure (vs. no Heart failure) | 1.07 (0.82-1.39) | 0.622 |
| Atrial fibrillation (vs. no Atrial fibrillation) | 1.17 (0.91-1.53) | 0.225 |
| Hypertension (vs. no Hypertension) | 0.59 (0.46-0.75) | < 0.001 |
| Hyperlipidemia (vs. no Hyperlipidemia) | 0.44 (0.34-0.56) | < 0.001 |
| Diabetes with complication  (vs. Diabetes without complication) | 0.71 (0.55-0.91) | 0.007 |
| COPD (vs. no COPD) | 0.78 (0.54-1.15) | 0.220 |
| CKD (vs. no CKD) | 1.12 (0.87-1.43) | 0.374 |
| Stroke (vs. no Stroke) | 1.48 (1.11-1.97) | 0.008 |
| WBC | 1.04 (1.03-1.06) | < 0.001 |
| RBC | 0.76 (0.64-0.89) | 0.001 |
| Platelet | 1.00 (0.99-1.00) | 0.822 |
| Hemoglobin | 0.89 (0.85-0.95) | < 0.001 |
| Hematocrit | 0.97 (0.96-0.99) | 0.014 |
| Potassium | 1.46 (1.25-1.71) | < 0.001 |
| Sodium | 0.99 (0.97-1.01) | 0.380 |
| Chloride | 0.99 (0.98-1.02) | 0.663 |
| Glucose | 1.00 (1.00-1.00) | 0.138 |
| RDW | 1.14 (1.09-1.19) | < 0.001 |
| Albumin | 0.58 (0.48-0.71) | < 0.001 |
| RAR | 1.32 (1.23-1.42) | < 0.001 |
| SOFA score | 1.08 (1.03-1.13) | 0.002 |
| SIRS score | 1.18 (1.03-1.36) | 0.017 |
| Antiplatelet drugs (vs. no Antiplatelet drugs) | 0.44 (0.33-0.57) | < 0.001 |
| Statins (vs. no Statins) | 0.41 (0.31-0.53) | < 0.001 |
| Insulin (vs. no Insulin) | 0.77 (0.44-1.36) | 0.373 |
| Mechanical ventilation (vs. no Mechanical ventilation) | 1.13 (0.88-1.46) | 0.340 |

SBP, systolic blood pressure; DBP, diastolic blood pressure; MBP, mean blood pressure; SpO2, pulse oxygen saturation; COPD, chronic obstructive pulmonary disease; CKD, chronic kidney disease; WBC, white blood cell; RBC, red blood cell; RDW, red blood cell distribution width; RAR, red blood cell distribution width-to-albumin ratio; SOFA, Sequential Organ Failure Assessment; SIRS, Systemic inflammatory response syndrome.

**Supplementary Table 3. Collinearity diagnostics for screening variables.**

| Characteristic | Tolerance | VIF |
| --- | --- | --- |
| Age | 0.805 | 1.243 |
| Race | 0.976 | 1.025 |
| SBP | 0.328 | 3.052 |
| DBP | 0.152 | 6.585 |
| MBP | 0.099 | 10.057 |
| Temperature | 0.963 | 1.039 |
| Hypertension | 0.910 | 1.099 |
| Hyperlipidemia | 0.939 | 1.065 |
| Diabetes with complication | 0.889 | 1.124 |
| Stroke | 0.963 | 1.038 |
| WBC | 0.919 | 1.088 |
| RBC | 0.144 | 6.922 |
| Hemoglobin | 0.059 | 16.841 |
| Hematocrit | 0.042 | 23.639 |
| Potassium | 0.938 | 1.066 |
| SOFA score | 0.901 | 1.110 |
| SIRS score | 0.901 | 1.109 |
| Antiplatelet drugs | 0.782 | 1.278 |
| Statins | 0.804 | 1.244 |

VIF, variance inflation factor; SBP, systolic blood pressure; DBP, diastolic blood pressure; MBP, mean blood pressure; WBC, white blood cell; RBC, red blood cell; SOFA, Sequential Organ Failure Assessment; SIRS, Systemic inflammatory response syndrome.

**Supplementary Table 4. Comparison of differences between AUCs in ROC curve analysis.**

| Categories | AUC differences | P-value |
| --- | --- | --- |
| 1-year mortality | | |
| RAR-RDW | 0.03 | 0.015 |
| 30-day mortality | | |
| RAR-RDW | 0.03 | 0.047 |
| 90-day mortality | | |
| RAR-RDW | 0.04 | 0.012 |

AUC, area under the curve; RAR, red blood cell distribution width-to-albumin ratio; RDW, red blood cell distribution width.
